# Barriers and Facilitators to Intradialytic Parenteral Nutrition Implementation Targeting Protein Energy Wasting in Malaysian Hemodialysis Patients

**DOI:** 10.3390/healthcare10102090

**Published:** 2022-10-20

**Authors:** Birinder Kaur Sadu Singh, Ban-Hock Khor, Sharmela Sahathevan, Abdul Halim Abdul Gafor, Enrico Fiaccadori, Karuthan Chinna, See-Hoe Ng, Tilakavati Karupaiah

**Affiliations:** 1 Department of Pharmacy, Hospital Canselor Tuanku Muhriz, Universiti Kebangsaan Malaysia, Kuala Lumpur 56000, Malaysia; 2 Faculty of Food Science and Nutrition, Universiti Malaysia Sabah, Kota Kinabalu 88400, Malaysia; 3 Division of Nutrition and Dietetics, Faculty of Health Sciences, International Medical University, Kuala Lumpur 57000, Malaysia; 4 Department of Medicine, Hospital Canselor Tuanku Muhriz, Universiti Kebangsaan Malaysia, Kuala Lumpur 56000, Malaysia; 5 Nephrology Unit, Department of Medicine and Surgery, Parma University Hospital, 43121 Parma, Italy; 6 Faculty of Business and Management, USCI University, Kuala Lumpur 56000, Malaysia; 7 School of BioSciences, Faculty of Health and Medical Sciences, Taylor’s University Lakeside, Selangor 47500, Malaysia

**Keywords:** intradialytic parenteral nutrition, protein energy wasting, hemodialysis, PARIHS framework, implementation

## Abstract

The capacity to deliver intradialytic parenteral nutrition (IDPN) for patients on hemodialysis (HD) diagnosed with protein energy wasting (PEW) in low resource settings is unknown. This study aimed to examine the extent of IDPN practice in HD units in Malaysia, and its implementation to treat PEW. We surveyed pharmacists (*n* = 56), who are central to parenteral nutrition delivery in Malaysia including IDPN. Seventeen healthcare stakeholders engaging with the Promoting Action on Research Implementation in Health Services (PARIHS) framework used the Likert scale to rate survey outcomes on IDPN implementation to treat PEW, according to the *Evidence*, *Context*, and *Facilitation* elements. IDPN for HD patients was available in 28 of 56 hospitals providing parenteral nutrition services, with only 13 hospitals (23.2%) providing IDPN to outpatients. Outpatient treatment was concentrated to urban locations (12/13) and significantly associated (*p* < 0.001) with resident nephrologists. The *Evidence* domain was rated poorly (2.18 ± 0.15) pertaining to IDPN indication when the oral spontaneous intake was ≤20 kcal/kg/day. The *Context* domain indicated good adherence to international best practice relating to IDPN administration (4.59 ± 0.15) and infusion time (4.59 ± 0.12). Poor adherence was observed in the *Facilitation* domain on ’Access to pharmacist and dietitian at HD units’ (2.65 ± 0.21) and ’Access to continuous medical education on managing PEW patients on HD’ (2.53 ± 0.15). The IDPN outpatient service was concentrated to urban hospitals with greater manpower resources. The PARIHS evaluation on IDPN implementation to treat PEW revealed facilitators in good practice adherence for prescribing and administration of IDPN but highlighted major barriers relating to IDPN indication and nutrient calculation.

## 1. Introduction

The Global Nutrition Care Atlas Survey highlights challenges in many low-middle income countries (LMICs) with regards to the prescribing of oral nutritional supplementation (ONS) to treat protein energy wasting (PEW) [1]. PEW is a severe form of malnutrition [2], variously prevalent between 28% and 54% globally across 34 countries [3]. The concern is PEW constitutes a strong predictor of mortality in chronic kidney disease (CKD) patients. Experts view that PEW patients are unable to meet nutritional needs with diet alone due to underlying anorexia, systemic inflammation, and auto-immune conditions [4,5] contributing to suboptimal dietary energy (DEI) and protein intakes (DPI) [6]. The International Society of Renal Nutrition and Metabolism (ISRNM) treatment algorithm [5] supports nutritional optimization through nutrition support to correct nutrient deficits with the choice of either oral or parenteral routes.

Of importance, the professional capacity to administer nutrition treatment given the backdrop of dietitian shortages in many low resource settings [7] is unknown. Malaysia, an LMIC with documented low access to nutrition care [8], has a PEW prevalence of 23.1% in hemodialysis (HD) patients [9]. Although ONS for PEW treatment in Malaysia has borne positive outcomes on muscle wasting [10], this was achieved with a research dietitian. In contrast, opting for intradialytic parenteral nutrition (IDPN) intervention to treat PEW [11] if ONS fails to correct DEI and DPI deficits as per the ISRNM treatment algorithm [5] has not been trialed in a low resource setting such as Malaysia. IDPN practice is unknown in Malaysia except in acute care settings in hospitals [12,13], and its use is based on medical opinion without specific practice guidelines to guide the delivery of IDPN intervention to HD patients [13]. Therefore, an evaluation on the current practice of IDPN is timely to assess the professional capacity to deliver IDPN to HD patients with overt PEW.

The Promoting Action on Research Implementation in Health Services (PARIHS) framework [14], unlike other theoretical models in implementation science [15], enables a defined process for implementing a health service, facilitates diagnostic analysis of framework elements [16], and aids in selecting appropriate implementation strategies and measurement for successful outcomes [17]. It’s interactive elements comprise *Evidence*, which references varied sources of knowledge and information combined and utilized for clinical decision making; *Context*, which demonstrates the implementation of *Evidence* into practice by defining the environment or setting in which the research is implemented; and *Facilitation*, which defines the implementation process of *Evidence* into practice by engaging facilitators with appropriate skills, roles, and knowledge to help individuals, teams, and organizations [14].

We applied a mixed methods approach to first quantitatively study the extent of IDPN practice in Malaysia through a survey. Pharmacists were targeted as respondents as they are traditionally tasked with parenteral nutrition services including IDPN in Malaysia [11,12]. We then qualitatively evaluated their practice by adopting the PARIHS framework model to facilitate a diagnostic analysis of survey outcomes and generate recommendations for improving IDPN delivery in Malaysia benchmarked to international practice standards. Outcomes from this study are expected to guide the implementation of IDPN practice in outpatient settings to treat PEW in Malaysian HD patients.

## 2. Materials and Methods

### 2.1. Study Design

This study adopted the mixed methods approach with Phase I’s cross-sectional survey on IDPN practice, and Phase II examining the implementation of IDPN in the context of PEW treatment using the PARIHS framework.

### 2.2. Phase 1: Cross-Sectional Survey

#### 2.2.1. Sampling

The survey targeted registered pharmacists specifically identified as providing parenteral nutrition (PN), and renal pharmacy services at both Malaysian government and private hospitals. This information was sought directly through the National Pharmacy Services Department or through their website. Sixty-five pharmacists were thus identified with the criteria of practicing at least 12 months with one response per hospital practice obtained. The data collection was conducted via email and telephonic conversations between August and December 2018.

#### 2.2.2. Questionnaire Development

As studies relating to IDPN practice at outpatient HD units were scarce, we developed a questionnaire specific to determining the current status of IDPN delivery at outpatient HD units. We referenced studies reporting on PN practice delivery in hospitals, which allowed modelling some query items to IDPN practice [11,18] to suit the PARIHS framework elements. In addition, questions seeking criteria application for the PEW diagnosis were referenced to the ISRNM diagnostic criteria of body mass index (BMI) < 23 kg/m^2^, serum albumin < 38 g/L, weight loss of 10% over 6 months, and dietary intake < 25 kcal/kg body weight [2].

The questionnaire was reviewed for content validity by stakeholders in IDPN practice and HD patient care providers instead of pilot testing the questionnaire, owing to the small sampling frame of pharmacists engaged with PN delivery in Malaysia.

The finalized questionnaire consisted of 25 questions covering five domains as briefly described:

- Section A elicited four questions targeting sampled hospitals and HD units relating to demographic characteristics of the service provider, number of patients receiving HD treatment, access to nephrologists, and IDPN prescriptions in outpatient HD units.
- Section B elicited seven questions targeting the type of patients receiving IDPN, who initiates IDPN, and the preferred type of IDPN bag.
- Section C yielded eight questions focusing on IDPN prescription and administration.
- Section D included three questions on monitoring and evaluation of IDPN treatment of patients.
- Section E included three questions on the pharmacist’s role and tasks in IDPN delivery.

The questionnaire is available as Supplementary Material.

### 2.3. Phase 2: Evaluating Survey Outcomes

Survey outcomes on IDPN delivery practice from Phase 1 were subjected to a process workshop based on the PARIHS framework [14]. The steps involved were as follows.

#### 2.3.1. Evidence for Rating

Survey questions yielded practice-related evidence for 13 indicators relevant to the implementation of best practice in IDPN delivery for HD outpatient settings categorized according to the PARIHS framework model elements, namely *Evidence*, *Context*, and *Facilitation*; and benchmarked to available international IDPN best practice guidelines [19,20,21,22]. Table 1 summarizes the context of survey outcomes related to the three elements of PARIHS with the benchmarked standards for practice.

#### 2.3.2. Sample Recruitment for the SIS-ER Workshop

The State of IDPN Services-Expert Rating (SIS-ER) workshop convened an expert panel to examine survey evidence for indicators related to the domains of *Evidence*, *Context*, and *Facilitation*. Patient care providers involved in HD delivery were considered experts with reference to their clinical experience, management of IDPN patients at hospitals’ HD units, and research and publications pertaining to CKD. Experts were identified through pharmacist participants in Phase 1, with recruitment extended by the snowballing technique [23]. Panel diversity was achieved with representation from academia, public institutions, and HD units with the involvement of relevant multidisciplinary professions. Inclusion criteria included a minimum three years of experience in the relevant profession and being willing to participate in the rating process.

#### 2.3.3. Terms of Reference

Experts consenting to participate in the SIS-ER were emailed advance reading material on IDPN delivery and practice two weeks prior to the workshop. Their demographic data for age, gender, ethnicity, professional background, and years of working experience of experts were collected.

#### 2.3.4. Rating Process

Rating during the SIS-ER was performed for survey evidence on IDPN practice relating to the 13 indicators benchmarked to international best practice guidelines for IDPN practice [19,20,21,22]. The voting process for each question was conducted using an online rating program (www.mentimeter.com, accessed on 30 August 2022), with a common code for access provided to the mobile devices of the experts. This response tool ensured anonymity during SIS-ER. A 5-point Likert scale was applied to rate adherence to benchmarks, where ‘1′ indicated very poor, 2 as ‘poor’, 3 as ‘moderate’, 4 as ‘good’, and 5 as ‘very good’ adherence. Each expert also received a personalized rating form to manually record scores and comments.

**Figure 1:**
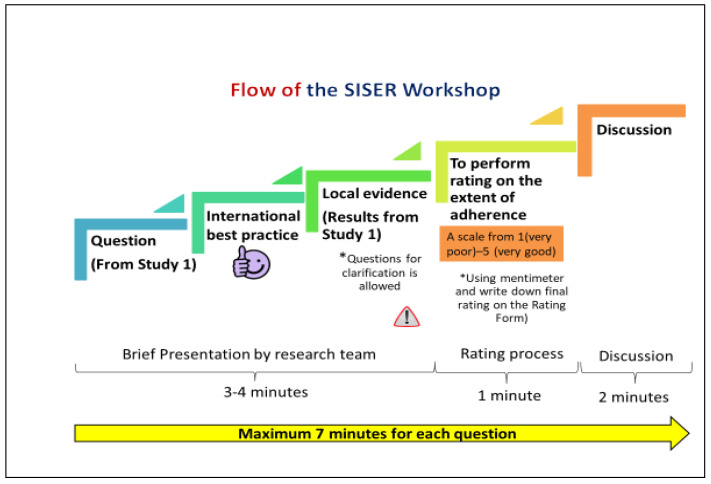
Process Flow from Question to Discussion in the SIS-ER Workshop.

The rating process adopted for the SIS-ER workshop followed the Food-Environment Policy Index Expert Rating (FEER) workshop process as reported by Ng et al. (2018) [24]. The flowchart of the SIS-ER workshop process is shown in Figure 1. The online voting results generated for each question, were captured and saved in Microsoft Excel format for data analysis. In addition, the SIS-ER workshop was recorded in video format to track any missing information during the data analysis.

### 2.4. Statistical Analysis

The survey analysis was primarily descriptive for categorical variables. A Chi-square analysis was applied to bivariate comparisons of pharmacists’ responses at hospitals with and without IDPN practice. SIS-ER outcomes for the related PARIHS element questions were interpreted as mean ± SE of Likert scores for overall expert feedback and by individual professional groups. Implementation of best practice in IDPN delivery aimed to achieve “good adherence” (Likert scale score 4–5) towards areas of practice benchmarked against international best practice guidelines, whilst scores < 3 were categorized as “poor adherence” and 3 to 4 as “moderate adherence”. Indicator scores rated as “good adherence” as per the professional group were converted into percentages. Statistical significance was set at *p* < 0.05. All analyses were computed using SPSS version 26.0 (IBM SPSS Statistics Inc. Chicago, IL, USA).

## 3. Results

### 3.1. Status of IDPN Delivery

#### 3.1.1. Demographics of IDPN Practice

Sixty-five pharmacists were identified and contacted, six declined to participate in the survey, while three hospitals did not have an HD unit; therefore, only fifty-six hospital pharmacists were eligible for respondent inclusion, yielding a response rate of 86.2%.

The provision of IDPN practice at HD units is presented in Table 2a,b. IDPN service was available at 28 hospitals but only 13 hospitals extended service to outpatient HD settings. The majority of hospitals (61.5%) offering IDPN practice were urban (*p* = 0.009), whereas the location of outpatient HD settings was not significantly different (*p* > 0.05). By sector distribution, government hospitals (80.4%) were the main IDPN providers. Most participating hospitals’ HD units (46.4%) had the capacity for dialyzing 50 to 100 HD patients per day. The majority of HD units (69.6%) had access to a visiting nephrologist with 30.4% having two or more resident nephrologists. Of note, the number of resident nephrologists in hospitals was significantly associated (*p* < 0.001) with the provision of IDPN practice in the outpatient HD units.

#### 3.1.2. Best Practice Indicators for IDPN Prescription (*Evidence*)

Pharmacists’ response to the adoption of international best practice guidelines regarding selection criteria to initiate IDPN for malnourished HD patients is shown in Table 2a. Most pharmacists (84.6%) reported nephrologists relied on serum albumin and dietary intake criteria to justify IDPN initiation. However, only 46.2% reported initiating IDPN for patients consuming 20 kcal/kg/day or less of their spontaneous oral intake.

#### 3.1.3. Leadership in Clinical Decision Making (*Context*)

Most respondents (77%) reported nephrologists were the primary decision makers for prescribing IDPN based on their subjective assessment of the patient’s serum albumin, dietary intake, and weight status (Table 2a). Although the doctor and pharmacist team (46%) selected the type of IDPN bag for the patient, the utilization of standard formulas (50.0%) superseded the practices of manually calculating macronutrients and adjusting fluid volume. Almost all HD units did not have a standard prescribing protocol for IDPN patients. Pharmacists (37.5%) determined the macronutrient doses at these units. The standard bags (69.2%) were the preferred choice for the prescribers. Less than 10% of the pharmacists compounded IDPN bags.

#### 3.1.4. Organization and Culture (*Context*)

All nurses and 23% of medical assistants were regularly involved in IDPN administration (Table 2a). The majority of HD units (92.3%) administered IDPN within the dialysis session lasting four hours. Typically, no infusions were given concurrently with IDPN (46.2%), whereas blood products (23.1%), intravenous antibiotics (15.4%), and saline (7.7%) were administered during IDPN infusion. Most infusions (84.6%) were not infused via the same intravenous line for IDPN administration.

#### 3.1.5. Roles, Tasks, and Performance in IDPN Delivery (*Facilitation*)

Table 2a also indicated that hospitals HD units had access to pharmacists and/or dietitians (76.9%). Although 76.9% of pharmacists were aware of PEW in CKD patients, more than half the pharmacists (53.8%) were unsure about the IDPN indication for PEW treatment. Almost 54% of pharmacists reported not having any access to continuous medical education on managing PEW patients.

### 3.2. SIS-ER Proceedings 

Seventeen out of twenty-four invited healthcare professionals working in dialysis services participated in the SIS-ER (70.8% response rate). This expert group comprised of nephrologists (*n* = 2), pharmacists (*n* = 6), dietitians (*n* = 5), and nurses/medical assistants (*n* = 4).

#### 3.2.1. Facilitators and Barriers to Good Practice

**Figure 2:**
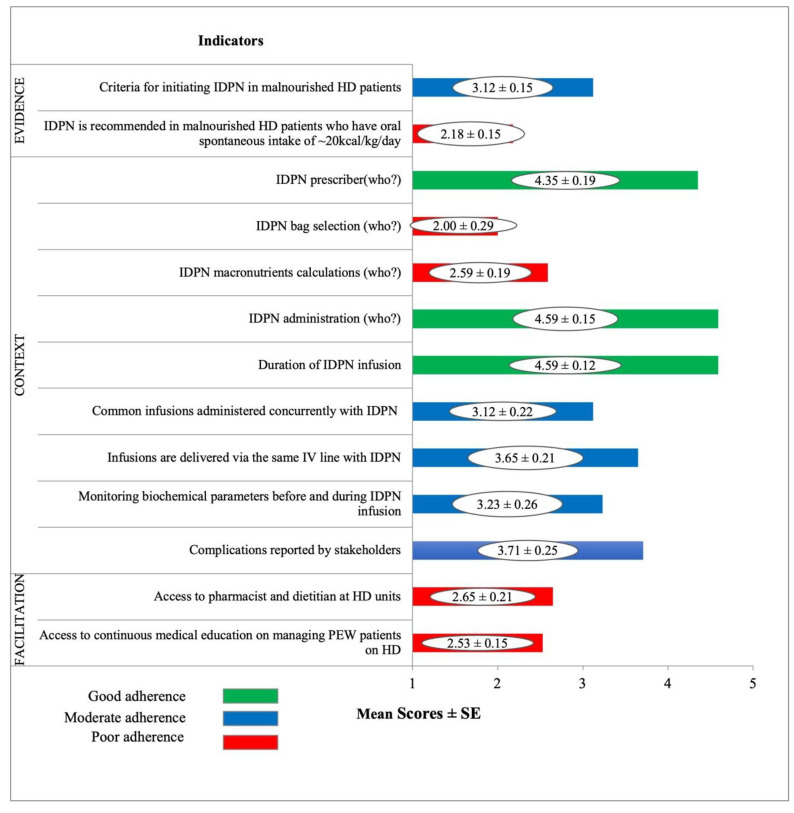
Mean ± SE Score Ratings from Experts as per Indicators and PARIHS Domains. Abbreviations: HD, Hemodialysis; IDPN, Intradialytic Parenteral Nutrition; IV, Intravenous; PEW, Protein Energy Wasting.

Figure 2 presents the mean ± SE scores for 13 indicators as per the PARIHS elements relevant to IDPN best practice guidelines. In total, 5 of 13 indicators were rated as poor adherence (mean score below 3.0), 5/13 indicators as moderate adherence, and 3/13 indicators as good adherence. Accordingly:

- In the *Evidence* domain, ‘*Criteria for initiating IDPN in malnourished HD patients*’ was rated as moderate adherence while IDPN is recommended in malnourished HD patients who have oral spontaneous intake of ~20 kcal/kg/day’ was rated as poor adherence.
- In the *Context* domain, three indicators relating to IDPN prescription, administration, and infusion duration were rated as good adherence. Moderately rated indictors related to sharing intravenous access, concurrent infusions, and monitoring of biochemical parameters and complication reporting. Bag selection and macronutrient calculation for IDPN were rated as poor adherence.
- In the *Facilitation* domain, ‘*Access to pharmacist and dietitian at HD unit*’ and ‘*Access to continuous medical education on managing PEW patients on HD*’ were rated as poor adherence.

#### 3.2.2. Ratings as per Profession

Expert ratings for PARIHS indicators were further examined based on the professional group with scores assessed as a percentage of good adherence to practice guidelines (Table 3).

Professional opinion diverged as regards sufficiency of the *Evidence* guiding IDPN practice. The IDPN practice indicator relating to ‘*Criteria for initiating IDPN in malnourished HD patients*’ was rated the poorest by dietitians, whilst 50% of nurses assigned a ‘good adherence’ rating to this indicator. However, pharmacists who rated poor adherence to best practice (17%) for this indicator, commented ‘IDPN should be resumed for outpatient HD patients if patients were on IDPN during hospitalisation’. Serum albumin and dietary intake were the only indicators confirming the decision to initiate IDPN, whereas an anthropometry assessment was not performed in the Malaysian setting. The dietitians commented ‘BMI and percentage of weight loss are important criteria, which can be evaluated by non-dietitians, but dietary intake (calorie and protein) should be assessed by a dietitian’.

With reference to the *Evidence* indicator ‘*IDPN is recommended in malnourished HD patients who have oral spontaneous intake of at least 20 kcal/kg/day*’, all professionals agreed that adherence to practice guidelines was poor (0%). The dietitians commented that ‘patient’s calorie intake assessment is critically required and can only be executed by dietitians with renal nutrition expertise’. They further added ‘It is important to refer newly dialyzed patients to a dietitian for dietary education to avoid suboptimal calorie and protein intakes’.

In terms of the *Context* domain related to IDPN practice, three of nine indicators reached consensus amongst the professional groups. Good adherence to practice guidelines (mean scores between 4.0 and 4.6) was rated as per the indicator ‘IDPN Prescriber’ by most experts irrespective of profession (75–100%). In Malaysia, doctors are authorized to write prescriptions. Therefore, only the nephrologist facilitates the IDPN prescription. Subsequently, all the professional groups strongly agreed for the indicators ‘*IDPN administration*’ and ‘*Duration of IDPN infusion*’ with mean scores ranging from (4.25 to 5.00) indicating good adherence to practice guidelines (75–100%) on duration of IDPN infusion in Malaysia, which was between 3.5 and 4 h.

**Figure 3:**
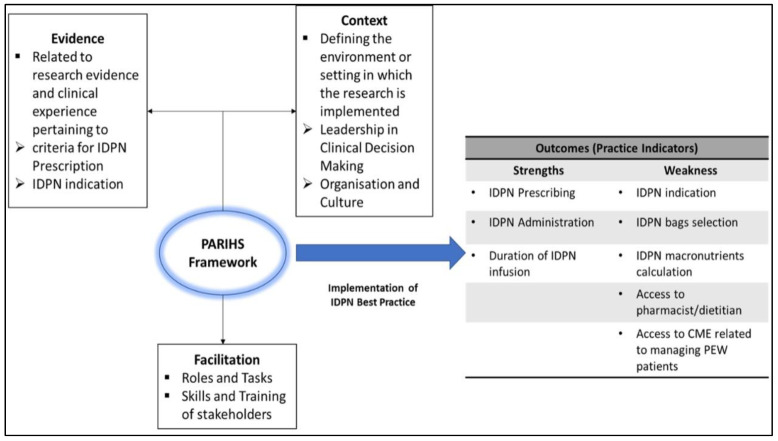
Outcome Summary of Implementation of IDPN Best Practice Defined by the PARIHS Framework.

However, specific to the IDPN bag selection indicator, nephrologists gave the lowest rating compared to other professions (Figure 3). In Malaysia, IDPN bag selection is decided by the nephrologist and pharmacist (refer to Table 2). The dietitians recommended that ‘Dietitians should be involved as a team member along with doctors and pharmacists for IDPN bag selection’.

Another *Context* indicator, ‘*IDPN macronutrients calculation’*, expectedly received the lowest rating from nephrologists as standard formula bags are preferred in Malaysia over individual compounding (refer to Table 2), indicating the likelihood of neglecting prescribing nutrients according to the patient’s metabolic status. The pharmacists commented that ‘There is a lack of dietitian involvement for IDPN in Malaysia”. The dietitians further stated that ‘A compounded PN bag based on patients’ requirement is ideal, but a standard PN bag is better than none’.

The *Facilitation* indicator ‘*Access to pharmacist and dietitian at HD units*’ was rated with poor adherence to practice guidelines by the doctors. Pharmacists also rated poor adherence to best practice (0%) for this indicator, commenting that ‘Both pharmacists and dietitians are needed in HD units’. For the *Facilitation* indicator ‘*Access to continuous medical education on managing PEW patients on HD*’, all professional groups except the doctors indicated poor adherence to practice guidelines (0%). Additionally, the pharmacists acknowledged that ‘Continuous Pharmacists Education is required on managing PEW patients on HD’.

Figure 3 summarizes the outcomes of the PARIHS framework assessment on the implementation of IDPN practice, highlighting strengths in three areas of IDPN practice as per IDPN prescribing, administration, and duration of infusion were following best practice standards. However, five weaknesses were highlighted relating to PEW treatment, namely IDPN indication, bag selection, macronutrient calculation, access to pharmacists/ dietitians, and continuous education.

## 4. Discussion

This study describes implementation issues related to IDPN service delivery to treat PEW in HD patients as recommended by the ISRNM [5] in Malaysia, which represents a low resource setting as per access to optimal kidney nutrition care [7]. In this mixed methods approach, we applied the survey findings of IDPN practice in Malaysia to evaluate the implementation capacity for PEW treatment with the PARIHS. The PARIHS provides a tool-based framework enabling healthcare practitioners to understand the complexity of implementing a health service and the elements that require attention for implementation to be successful [14], and it has been applied to healthcare practices involving the development of survey instruments [25] and implementation of evidence-based nursing care practice [26]. The IDPN practice in Malaysia was concentrated in urban hospitals. The prescription of IDPN was more likely in public than private hospitals with their HD units having access to nephrologists [27]. IDPN services were more often provided for general wards or acute care settings than in the outpatient settings. A key survey finding was IDPN prescription in outpatient HD settings was directly associated with the number of resident nephrologists (*p* < 0.001). This is similar to the United States, where IDPN prescriptions are also provided by physicians [11], whereas in Australia, the responsibilities lie with the dietitian and/or the treating team [28].

A critical question was whether this current IDPN practice in Malaysia was suitable to deliver PEW treatment. Implementation issues were examined qualitatively using the PARIHS framework [14] and the adoption of its three core elements, namely, *Evidence*, *Context*, and *Facilitation*. The outcomes indicated more weaknesses than strengths in the IDPN service delivery to tackle PEW treatment. Related to the Evidence context, our finding was IDPN provision was occurring without an oral intake assessment. Further, suboptimal oral intake below 20 kcal/kg/day was not established for Malaysian HD patients, which justifies the provision of parenteral nutrition [21]. The additional justification to treat PEW patients through IDPN is to provide 25% of the total targeted nutrient intake with the remaining from the patient’s usual diet [21]. Optimizing dietary energy and protein intakes will mitigate the negative nitrogen balance, the iatrogenic amino acid losses incurred through the dialysis procedure, and dietary inadequacy from anorexia and poor appetite associated with PEW patients [6].

Cross-cutting implementation issues understood from the two *Context*-related indicators, ‘*IDPN bag selection*’ and ‘*IDPN macronutrients calculation*’ emphasized the lack of professional involvement of the dietitian. Firstly, IDPN bag selection was only decided by nephrologists and pharmacists, unlike in the United Kingdom [19], Australia [28], and the United States [11] where the renal dietitian and nephrologist jointly decide on the IDPN regime selection. Secondly, ‘*IDPN macronutrients calculation*’ appeared to solely depend on the pharmacist following the usual practice for parenteral nutrition provision in Malaysian hospital settings [12]. This contrasts with the United Kingdom’s NHS IDPN Guidelines (2018) [19] and Canada’s BC Renal Agency IDPN Guidelines (2019) [20] suggesting the requirement for a renal dietitian to calculate and provide an individualized IDPN regime. The lack of professional consultation to perform macronutrient calculation is reflective of the ‘silo’ practice [29], which limits personalized compounding necessary to optimize nutritional requirements for PEW patients. This also increased the dependency of pharmacists in Malaysia on the ’one size fits all’ standard 3-chamber IDPN bag formulations as indicated by the survey. The disadvantages of standard 3-chamber IDPN bags included their low protein content in high volume bags [30], which were insufficient to meet HD patient needs. 

A further barrier limiting IDPN provision to treat PEW in outpatient settings was poor access to professional support as indicted by the *Facilitation* indicator because of insufficient access to full-time pharmacists (15.4%) and dietitians (7.7%). This calls for an organization-wide change [29] in order to optimize IDPN service in a non-traditional setting to treat HD patients with PEW. Interprofessional collaboration with face-to-face interactions and regular communication have been observed to improve patient-centered care in HD units [31]. The dietitian accessibility in Malaysia was only 32.7% at HD units [8], confined to urban hospitals, and concentrated in government settings. The lack of routine involvement of dietitians at HD units in Malaysia [8] also reflects the prevailing scenario of limited renal dietitian services in many other countries [7]. In contrast, it is mandatory in the United States for dialysis facilities to have a dietitian member in the multidisciplinary team for patient care, while dialysis facilities are encouraged to use the pharmacist’s expertise as appropriate [32].

The successful implementation of IDPN provision to treat PEW in the outpatient HD setting will also benefit from the healthcare team’s access to CMEs related to PEW. The *Facilitation* indicator was poorly rated as only 46% of pharmacists had attended related CMEs. Knowledge on the potential benefits of IDPN is crucial to raise conviction for its use when clinically indicated [28]. Furthermore, training on IDPN for dietitians and medical staff on the practicalities of patient selection for IDPN, choice of formulation, rate of delivery, and monitoring procedures will be useful [28].

The facilitators in IDPN delivery to treat PEW in Malaysian settings related to the stakeholder competencies in IDPN prescription, administration, and duration of IDPN infusion. Good adherence by professional groups to best practice guidelines were observed with two *Context* indicators relating to IDPN prescription and administration. Nurses were required to administer IDPN within 3.5 to 4 h as recommended by the NHS IDPN Guidelines (2018) [19] and the BC Renal Agency IDPN Guidelines (2019) [20]. Therefore, nurses in Malaysia having the experience and training to deliver parenteral nutrition in critical care settings were able to cope with IDPN administration [12]. IDPN prescription was well-facilitated by either the nephrologist alone or sometimes by a nephrologist and pharmacist team as indicated by the survey.

The major strength of this study is for the first time the PARIHS framework was applied to assess implementation of IDPN to treat PEW in an LMIC country such as Malaysia. This study’s limitation was pharmacists were the only stakeholder in the multidisciplinary team surveyed. Additionally, the calories and amount of nutrients prescribed by stakeholders were not included in the survey. However, we recognized that pharmacists played the primary role in IDPN delivery in Malaysia, and the National Pharmacy Board of Malaysia enabled adequate sampling and the regional representation of pharmacists engaged in IDPN services. The doctors’ representation in the expert panel was the smallest compared to other professional groups, which may limit their interpretations of the survey evidence for rating.

## 5. Conclusions

Overall, this mixed methods study revealed the profiling of IDPN practice in Malaysia. The typical organizational structure of IDPN services was the provision at HD settings for inpatients rather than outpatients, and availability to urban hospital HD units with resident nephrologists. The PARIHS framework approach revealed good practice adherence in the prescribing and administration of IDPN. However, with regards to PEW treatment for HD patients, barriers to implementation issues were related to the IDPN indication and nutrient calculation, which required the professional participation of the dietitian.

## Figures and Tables

**Table 1:** IDPN Best Practice Indicators According to PARIHS Domains.

Domain	Related Q	Related QA-Section	Best Practice Indicators
*Evidence* derived from knowledge-based sources which include research, clinical and patient experience, and information from local context [14]	Q12	SECTION C-IDPN Prescription and Delivery	Criteria for initiating IDPN in malnourished HD patients
	Q13	SECTION C-IDPN Prescription and Delivery	IDPN is recommended in malnourished HD patients who have oral spontaneous intake of ~20kcal/kg/day
*Context* refers to the environment or setting in which the proposed change is to be implemented and has, further, three sub-elements which include organizational culture, leadership, and evaluation [14]	Q6	SECTION B- IDPN Use by Prescribers and Stakeholders	IDPN prescriber
	Q7	SECTION B- IDPN Use by Prescribers and Stakeholders	IDPN bag selection (who?)
	Q15	SECTION C-IDPN Prescription and Delivery	IDPN macronutrients calculations (who?)
	Q16	SECTION C-IDPN Prescription and Delivery	IDPN administration (who?)
	Q17	SECTION C-IDPN Prescription and Delivery	Duration of IDPN infusion
	Q18	SECTION C-IDPN Prescription and Delivery	Common infusions administered concurrently with IDPN
	Q19	SECTION C-IDPN Prescription and Delivery	Infusions are delivered via the same IV line with IDPN
	Q20 & 21	SECTION D- IDPN Monitoring and Evaluation of Treatment	Monitoring biochemical parameters before and during IDPN infusion
	Q22	SECTION D- IDPN Monitoring and Evaluation of Treatment	Complications reported by stakeholders
*Facilitation* refers to the method to simplify things for others through support and assistance in changing their attitudes, habits, skills, thinking process, and working [14]	Q23	SECTION E-Pharmacist’s Role and Tasks in IDPN Delivery	Access to pharmacist and dietitian at HD units
	Q25	SECTION E-Pharmacist’s Role and Tasks in IDPN Delivery	Access to continuous medical education on managing PEW patients on HD

Abbreviations: HD, Hemodialysis; IDPN, Intradialytic Parenteral Nutrition; Q, Question; QA, Questionnaire.

**Table 2:** Survey Outcomes on IDPN Practice at Malaysian Hospitals and Factors Affecting IDPN Practice at Outpatients’ HD units.

(a) Survey Outcomes on IDPN Practice at Malaysian Hospitals.								
				Hospitals with PN Service (*n* = 56) (*n*, %)		Hospitals Providing IDPN at Outpatient HD Units (*n* = 13) (*n*, %)		
*Type of Hospital*								
Government				45 (80.4)		12 (92.3)		
Private				11 (19.6)		1 (7.7)		
NGO				0 (0.0)		0 (0.0)		
*Location of Hospitals*								
Urban				39 (69.6)		12 (92.3)		
Rural				17 (30.4)		1 (7.7)		
*Frequency of Outpatients on HD*								
Less than 50				17 (30.4)		4 (30.8)		
50–100				26 (46.4)		6 (46.1)		
More than 100				13 (23.2)		3 (23.1)		
*Frequency of Nephrologist’s Access*								
1				39 (69.6)		3 (23.1)		
2 or more				17 (30.4)		10 (76.9)		
Best Practice Indicators for IDPN Prescription (*n* = 13)								
*Criteria for Initiating IDPN*								
Body mass index (BMI) < 23				7 (53.8)				
Serum albumin < 38 g/L				11 (84.6)				
Weight loss of 10% over 6 months				6 (46.2)				
Dietary intake < 25 kcal/kg BW				11 (84.6)				
*Pharmacist Recommendation to Initiate IDPN for Patients with at least 20 kcal/kg/day of Spontaneous Oral Intake?*								
Yes				6 (46.2)				
No				7 (53.8)				
Leadership in Clinical Decision Making (*n* = 13)								
*IDPN Prescribed By*								
Doctor only				10 (76.9)				
Doctor and pharmacists				3 (23.1)				
*IDPN Bag Selected By*								
Doctor only				3 (23.1)				
Pharmacist only				4 (30.8)				
Doctor and pharmacist				6 (46.1)				
*Who Calculates the IDPN Macronutrients*? *								
Doctor				1 (6.25)				
Pharmacist				6 (37.5)				
Dietitian				1 (6.25)				
Standard formula used				8 (50.0)				
*Type of IDPN Bags Supplied*								
Compounded bags by hospital pharmacy				1 (7.7)				
Standard bags				9 (69.2)				
Combination compounded and standard bags				3 (23.1)				
*IDPN Prescribing Protocol Availability*								
Yes				0 (0.0)				
No				13 (100.0)				
Organization and Culture (*n* = 13)								
*Staff Responsibility for IDPN Administration* *								
Doctor				1 (7.7)				
Nurse				13 (100.0)				
Medical Assistant				3 (23.1)				
Dietitian				0 (0.0)				
Pharmacist				0 (0.0)				
*IDPN Infusion Time*								
3.5 h or less				1 (7.7)				
4 h				12 (92.3)				
*Infusions Administered Concurrently with IDPN* *								
IV saline				1 (7.7)				
IV antibiotics				2 (15.4)				
Blood products				3 (23.1)				
No infusions				6 (46.2)				
*Are Infusions Given via the Same IV Line with IDPN*?								
Yes				2 (15.4)				
No				11 (84.6)				
Roles, Tasks, and Performance of Pharmacists (*n* = 13)								
Access to Supporting Staff *								
Full time pharmacist only				2 (15.4)				
Full time dietitian only				1 (7.7)				
Both pharmacist and dietitian				7 (53.8)				
No access				3 (23.1)				
*Is the Pharmacist Aware About PEW in Chronic Kidney Failure Patients?*								
Yes				10 (76.9)				
No				3 (23.1)				
*Access to CME on Managing PEW Patients on HD*								
Yes				6 (46.2)				
No				7 (53.8)				
**(b) Factors Affecting IDPN Practice at Outpatient HD Units.**								
**Characteristics**	**IDPN for all Patients (*n*,%)**				**IDPN for Outpatients (*n*,%)**			
	***n***	**Yes**	**No**	***p*** **-Value ^a^**	***n***	**Yes**	**No**	***p*** **-Value ^a^**
Urban	39	24 (61.5)	15 (38.5)	0.009	24	12 (50.0)	12 (50.0)	>0.05
Rural	17	4 (23.5)	13 (76.5)		4	1 (25.0)	3 (75.0)	
Nephrologist Availability								
1	39	14 (35.9)	25 (64.1)	0.001	39	3 (7.7)	36 (92.3)	<0.001
2 or more	17	14 (82.4)	3 (17.6)		17	10 (58.8)	7 (41.2)	

Abbreviations: BMI, Body Mass Index; BW, Body Weight; CME: Continuous Medical Education; HD, Hemodialysis; IDPN, Intradialytic Parenteral Nutrition; IV, Intravenous; NGO, Non-Government Organization. * More than one answer. ^a^ *Chi-square* analysis was used for categorical variables with *p* < 0.05 as significant.

**Table 3:** Percentage and Mean Scores Rated by Individual Health Professional Group with Good Adherence to Practice Guidelines.

No	Indicators	Dietitian (*n* = 5)	Doctor (*n* = 2)	Nurse (*n =* 4)	Pharmacist (*n =* 6)
1	Criteria for initiating IDPN in malnourished HD patients	20% (2.80)	0% (3.00)	50% (3.50)	17% (3.17)
2	IDPN is recommended in malnourished HD patients with oral spontaneous intake of ~20 kcal/kg/day	0% (2.00)	0% (2.00)	0% (2.00)	0% (2.00)
3	IDPN prescriber	100% (4.60)	100% (4.00)	75% (4.00)	100% (4.50)
4	IDPN bag selection (who?)	40% (2.40)	0% (1.00)	0% (2.50)	0% (1.67)
5	IDPN macronutrients calculation (who?)	0% (2.40)	0% (1.00)	25% (2.50)	17% (1.67)
6	IDPN administration (who?)	100% (4.80)	100% (5.00)	75% (4.25)	100% (4.50)
7	Duration of IDPN infusion	100% (5.00)	100% (4.00)	100% (4.25)	100% (4.67)
8	Common infusions administered concurrently with IDPN	60% (3.20)	0% (2.00)	25% (3.00)	67% (3.50)
9	Infusions are delivered via the same IV line with IDPN	60% (3.60)	100% (4.00)	50% (3.25)	83% (3.83)
10	Monitoring of biochemical parameters before and during IDPN infusion	40% (3.40)	0% (2.50)	50% (3.75)	33% (3.00)
11	Complications reported by stakeholders	60% (3.80)	100% (4.00)	75% (3.75)	67% (3.50)
12	Access to pharmacist and dietitian at HD units	40% (2.60)	0% (2.50)	25% (2.75)	0% (2.67)
13	Access to continuous medical education on managing PEW patients on HD	0% (2.60)	50% (3.00)	0% (2.75)	0% (2.17)

Note: Results reported as percentage of good adherence (Likert scale score 4 and 5) of a professional group to a cited indicator with mean Likert scale score for the group in parenthesis. Mean Likert scale score: below 3, poor adherence; 3–4, moderate adherence; 4–5, good adherence. Abbreviations: HD, Hemodialysis; IDPN, Intradialytic Parenteral Nutrition; IV, Intravenous; PEW, Protein Energy Wasting.

## Data Availability

The datasets generated and analyzed for the current study are available from the corresponding author, T.K., upon reasonable request.

## Supplementary Materials

The following supporting information can be downloaded at: https://www.mdpi.com/article/10.3390/healthcare10102090/s1. Questionnaire: A Survey on Intradialytic Parenteral Nutrition (IDPN) Practice at Malaysian Hospitals Haemodialysis (HD) Centres
