# Supplementary material for: Barriers and Facilitators to Intradialytic Parenteral Nutrition Implementation Targeting Protein Energy Wasting in Malaysian Hemodialysis Patients
Source: Healthcare (Basel). 2022 Oct 20;10(10):2090. doi: 10.3390/healthcare10102090 (PMC9602673; doi:10.3390/healthcare10102090)
Supplement: Supplementary file 1 [file healthcare-10-02090-s001.zip › healthcare-1916881-supplementary.pdf]

A Survey on Intradialytic Parenteral Nutrition (IDPN) Practice at Malaysian Hospitals Haemodialysis (HD) Centres

Name:__________________________ Hospital: __________________________

A. Demographics of IDPN practice

1. Type of Hospital: ☐ Government ☐ University ☐ Private

2. Is there a Nephrologist (Renal Specialist) at your centre?
☐ Yes ☐ No

If Yes please state the number:____________________

3. Total number of patients at your HD centre?: ______________

4. Does the Nephrologist prescribe IDPN for outpatient HD patients at the hospital?
☐Yes ☐ No

B. IDPN Use by Prescribers and Stakeholders

May tick more than one.

5. Patients who are prescribed on IDPN by the doctor are usually
☐ warded patients
☐ outpatients

6. Who determines to start or initiate IDPN at your hospital?
☐ Nephrologist/Doctor
☐ Nurse
☐ Pharmacist
☐ Dietitian
☐ Others please specify__________________________

7. Who selects or determines the IDPN bag to be used in patients?
☐ Doctor ☐ Pharmacist ☐ Dietitian

8. IDPN bags are provided as
☐ compounded bags by hospital pharmacy
☐ standard 3 chamber bags
☐ lipid-free standard bags
☐ others please specify_____________________________

CKD, Chronic Kidney Disease; CRP, C-Reactive Protein; HD, Haemodialysis; PN, Parenteral Nutrition; IDPN, ICU, Intensive Care Unit; Intradialytic Parenteral Nutrition; IV, Intravenous; LCT, Long Chain Triglycerides;MCT, Medium Chain Triglycerides.

9. Which of the following IDPN bag is commonly prescribed for your patients?
   - ☐ Nutriflex lipid special
   - ☐ Kabiven Peri & central
   - ☐ Nutriflex lipid peri & plus
   - ☐ Smof Kabiven
   - ☐ Others please specify____________________________

10. Is compounding individualized IDPN bag common (≥50% of IDPN prescribed at the HD unit are compounded regimes) for patients at your centre?
    (If yes proceed to question 11 , if No proceed to question 12)
    ☐ Yes ☐ No

11. Why is compounding preferred compared to standard formula for IDPN bag?
    ____

C. IDPN prescription and delivery

May tick more than one.

12. Which of these markers are used as a criteria by the nephrologist before initiating IDPN?
    - ☐ BMI <23kg/m^2
    - ☐ serum albumin <38g/L
    - ☐ weight loss of 10% over 6 months
    - ☐ dietary intake < 25kcal/kg/day
    - ☐ others please specify _______________________________

13.IDPN is recommended when maintenance HD patients have at least 20kcal/kg/day of established oral spontaneous intake. **Is this true**?
   ☐ Yes ☐ No

14. Is there specific IDPN prescribing protocol in place at your centre?
    eg. specific IDPN order form ☐ Yes ☐ No

15. Who calculates the macronutrients (carbohydrate,protein and lipid) prescribed to patients on IDPN?
    - ☐ Nurse
    - ☐ Doctor
    - ☐ Pharmacist
    - ☐ Dietitian
    - ☐ No calculation, use standard formula

16. Who administers IDPN at your HD centre?
   ☐ Nurse
   ☐ Doctor
   ☐ Medical Assistant
   ☐ Patient
   ☐ Others please specify____________________________

17. How many hours is IDPN administered in HD patients at your centre?
   ☐ 3.5 hours or less
   ☐ 4 hours
   ☐ 6 hours
   ☐ 12 hours
   ☐ 24 hour
   ☐ Others please specify____________________________

18. What are other infusions commonly given concurrently with IDPN?
   ☐ IV saline
   ☐ IV Antibiotics (e.g. IV Vancomycin)
   ☐ IV Iron
   ☐ Blood products
   ☐ Insulin
   ☐ None
   ☐ Others please specify____________________________

19. Are there drug or blood products infusions given via the same IV line as IDPN?
   ☐ Yes ☐ No
   ☐ If yes, please specify drug/blood product given________________________

D. IDPN Monitoring & Evaluation of treatment

May tick more than one.

20. Are baseline biochemical parameters done **before commencing** IDPN at your centre? **(if yes, please proceed to question 21)**
   ☐ Yes ☐ No

21. Which are the routine biochemical test done on outpatient HD patients while on IDPN?

☐ Serum electrolytes	☐ Total cholesterol
☐ Liver function tests	☐ Serum triglycerides
☐ Renal function tests	☐ Total bilirubin
☐ Blood glucose levels	☐ Dextrostix
☐ Serum Albumin	☐ Hemoglobin

CKD, Chronic Kidney Disease; CRP, C-Reactive Protein; HD, Haemodialysis; PN, Parenteral Nutrition; IDPN, ICU, Intensive Care Unit; Intradialytic Parenteral Nutrition; IV, Intravenous; LCT, Long Chain Triglycerides;MCT, Medium Chain Triglycerides.

☐ CRP
☐ Serum Iron
☐ Fluid status

22. What are the common complications reported in IDPN patients.

☐ Fever
☐ Hypotension
☐ Electrolyte imbalance
☐ IV line infection
☐ Hepatic Steatosis
☐ Hypercholesterolemia
☐ Fluid overload
☐ Hypotermia
☐ Hyperglycemia
☐ Sepsis
☐ Thrombophlebitis
☐ Hypertriglyceridemia
☐ Refeeding Syndrome

E. Pharmacists role and tasks in IDPN delivery

May tick more than one.

23. Does the dialysis center have access to a pharmacist/dietitian?

☐ Yes, full time pharmacist only
☐ Yes, full time dietitian only
☐ Yes, both
☐ No

24.Are you aware of Protein Energy Wasting (PEW) in CKD patients?

☐ Yes ☐ No

25.Did you receive any formal training or continuous medical education on managing PEW patients on HD?

☐ Yes ☐ No

Thank you for your time!

CKD, Chronic Kidney Disease; CRP, C-Reactive Protein; HD, Haemodialysis; PN, Parenteral Nutrition; IDPN, ICU, Intensive Care Unit; Intradialytic Parenteral Nutrition; IV, Intravenous; LCT, Long Chain Triglycerides;MCT, Medium Chain Triglycerides.
